# Tripartite motif-containing protein 11 (TRIM11): a novel weapon against Alzheimer’s disease

**DOI:** 10.1038/s41392-023-01729-5

**Published:** 2024-01-01

**Authors:** Yichu Fu, Bi Zhang, Jianfeng Liu

**Affiliations:** College of Life Science and Technology, Key Laboratory of Molecular Biophysics of MOE, Huazhong University of Science and Technology, Wuhan, Hubei 430074 China

**Keywords:** Diseases of the nervous system, Preclinical research

In a recent study published in *Science*, Zhang et al. discovered that tripartite motif-containing (TRIM) protein 11 (TRIM11) exhibited strong inhibition on tau aggregation, which is a pathological characteristic of tauopathies including Alzheimer’s disease (AD).^1^ This makes TRIM11 a promising novel target for the treatment of AD and other tauopathies.

Tauopathies are characterized by intracellular neurofibrillary tangles consisting of hyperphosphorylated tau. AD belongs to secondary tauopathies with extracellular amyloid β (Aβ) plaques and is the most common form of neurodegenerative disease. Moreover, AD contributes to more than half of all dementia cases worldwide. Protein quality control (PQC) systems eliminate defective or excess tau proteins, while the gradual decline of PQC activity with age promotes the conversion of soluble tau proteins to fibrillar aggregates. However, the underlying mechanisms of this remain ambiguous. Recently, several TRIM proteins have been identified to be key components of the PQC systems. Nevertheless, it remains unknown whether and how those TRIM proteins participate in the development of tauopathies. The exceptional study performed by Zhang and colleagues creatively unraveled an invaluable capacity of TRIM11 in the prevention of tauopathies and systematically evaluated the potential of TRIM11 as a therapeutic target.

**Fig. 1:**
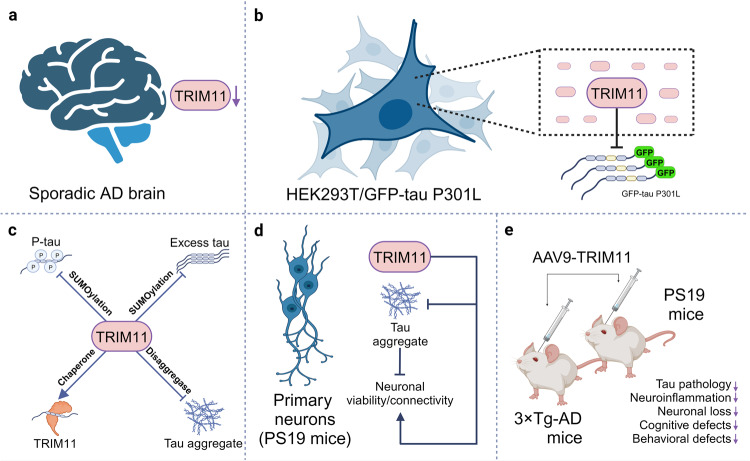
The role of TRIM11 on tau protein and tauopathies. Besides the fact that TRIM11 is down-regulated in the brain of AD patients (**a**), upregulation of TRIM11 greatly eliminates GFP-tau P301L aggregates in cultured cells (**b**). TRIM11 not only promotes the degradation of mutant/hyperphosphorylated tau and excess normal tau by binding to and SUMOylating them but also promotes tau solubility by serving as a molecular chaperone for tau and a disaggregate for tau aggregates/fibrils (**c**). Thus, TRIM11 would further inhibit the seeding of tau aggregates. Additionally, TRIM11 protects primary neurons against tau aggregation and accordingly maintains neuronal viability and connectivity (**d**). More importantly, TRIM11 ameliorates tauopathies in both PS19 and 3×Tg-AD mice (**e**). Figure was created with BioRender.com

In their study, the authors overexpressed and screened 75 TRIMs to remove GFP-tau P301L aggregates in cultured cells and found that TRIM11 exhibited the strongest effect (Fig. 1b). Further, they found that the level of TRIM11 protein was diminished during sporadic AD pathogenesis in postmortem brain tissues from AD patients (Fig. 1a). Both results strongly suggest that TRIM11 probably participates in tau pathology. Mechanistically, they first uncovered that TRIM11 promoted the proteasomal degradation of mutant/hyperphosphorylated tau and excess normal tau by directly binding to and SUMOylating them (Fig. 1c). Second, they provided evidence that TRIM11 would act as not only a molecular chaperone to inhibit the misfolding and aggregation of tau, but also a disaggregase to dissolve tau fibrils (Fig. 1c). This led to a decrease in tau aggregates and an increase in tau solubility. All these exceptional activities of TRIM11 would strongly maintain tau in its soluble state and prevent the progress of tauopathies.

Next, the authors found that TRIM11 was required to reduce tau pathology in primary cortical neurons isolated from PS19 mice (Fig. 1d). Silencing *TRIM11* expression with antisense oligonucleotides exacerbated tau aggregation, whereas upregulation of TRIM11 with an adeno-associated virus AAV9 delivery system (AAV9-TRIM11) had the opposite effects. Moreover, using genetic manipulation strategies, the authors discovered that TRIM11 was a strong neuroprotective factor that maintains neuron connectivity and viability by increasing neuronal integrity and synapse formation (Fig. 1d).

Then, hippocampal delivery of AAV9-TRIM11 greatly declined the levels of insoluble/soluble p-tau species and inhibited the formation of neurofibrillary tangles (NFTs)-like tau inclusions/tau-aggregations in PS19 mice, a common mice model mimicking human AD and other tauopathies. Moreover, AAV9-TRIM11 further reduced the hyperactivation of glial cells and improved cognitive and motor strength in these mice. Similar therapeutic effects were observed in accelerated disease phenotypes in PS19 mice inoculated with tau-preformed fibrils (PFFs) (Fig. 1e). In another transgenic AD model (3×Tg-AD mice), which displays a combination of Aβ plaques and tau tangles similar to human AD, both hippocampal and cerebrospinal fluid delivery of AAV9-TRIM11 reduced tau pathology, decreased hippocampal astrogliosis and microgliosis, and ameliorated cognitive impairment (Fig. 1e). All these results eminently demonstrates that AAV9-TRIM11 shows great potential in treating AD.

Collectively, TRIM11 is a multifunctional protein: it binds to and SUMOylates tau for degradation and maintains/promotes tau solubility as a chaperone and a disaggregase (Fig. 1). The functional multipotency of TRIM11 endows it with the capacity to prevent and treat tauopathies via multiple pathways simultaneously. As such, TRIM11 displays remarkable therapeutic potential as an anti-tauopathies weapon to fight AD and other neurodegenerative tau disorders. Importantly, applying AAV9 vectors to deliver TRIM11 into the hippocampi or whole brain regions through cerebrospinal fluid showed impressive efficacy in all mice models tested in their study. Since AAV-based gene therapy has been successfully translated into clinical treatments, AAV-mediated TRIM11 delivery might possess excellent potential in treating tau pathology in AD patients. Alternatively, mRNA- and protein-based platforms might also be suitable to deliver TRIM11, which may display a non-inferior effect with more safety.^2^ More interestingly, another study performed by the same group reported that TRIM11 could inhibit and reverse the formation of α-Syn fibrillar aggregates, subsequently mitigating neurodegeneration and motor defects in a mouse model of Parkinson’s disease.^3^ These results suggest that TRIM11 might be a powerful weapon to treat neurodegenerative diseases in a broader range.

It is worth mentioning that overexpression of TRIM11 may lead to tumor growth, migration, and invasion in glioma.^4^ The oncogenic feature of TRIM11 and its potential in AD treatment provide strong evidence that an imbalance of damage and repair drives disease and aging.^5^ Moreover, it should be extensively and critically evaluated whether upregulation of TRIM11 would present impressive efficacy in the prevention and treatment of AD in humans.
